# Lanthanide Metal-Organic Frameworks with Six-Coordinated Ln(III) Ions and Free Functional Organic Sites for Adsorptions and Extensive Catalytic Activities

**DOI:** 10.1038/srep29728

**Published:** 2016-07-19

**Authors:** Yu Zhu, Min Zhu, Li Xia, Yunlong Wu, Hui Hua, Jimin Xie

**Affiliations:** 1 College of Pharmacy and Chemistry & Chemical Engineering, Taizhou University, Taizhou, 225300, China; 2 Hanlin College, Nanjing University of Chinese Medicine, Taizhou, 225300, China; 3 School of Chemistry and Chemical Engineering, Jiangsu University, Zhenjiang, 212013, China

## Abstract

Three chelating-amino-functionalized lanthanide metal-organic frameworks, Y-DDQ, Dy-DDQ and Eu-DDQ, were synthesized with a flexible dicarboxylate ligand based on quinoxaline (H_2_DDQ = *N*, *N*′-dibenzoic acid-2,3-diaminoquinoxaline). The three-dimensional framework is constructed by the H_2_DDQ linkers connecting the zigzag ladders, showing a net of **sra** topology. In the structures, one kind of Ln(III) ions metal centers are six-coordinated and thus can potentially behave as open metal sites (OMSs), while the free chelating amino groups can act as free functional organic sites (FOSs). The N_2_ and Ar adsorption behaviors indicate that these Ln-DDQ exhibits stable microporous frameworks with high surface area after remove of the solvents. Owing to presence of OMSs and FOSs, these MOFs show good ability of CO_2_, dyes captures and Lewis acid catalyst for cyanosilylation reaction. In view of the existing FOSs in the framework, Pd NPs were immobilized onto the MOFs through graft interactions between free chelating amino groups and metal ions precursor using postsynthetic modification. The well dispersed Pd@Ln-DDQs exhibit efficient and recyclable catalytic reduction of 4-nitrophenol to 4-aminophenol, and they can also act as an excellent catalyst for Suzuki-Miyaura cross-coupling reactions with the exposed Pd NPs.

In the fast growing field of porous coordination materials, metal-organic frameworks (MOFs), is of great interest to achieve systematic functionalization of the pores for the applications in gas absorption^1^,^2^,^3^, catalysis^4^,^5^, chemical sensing^6^,^7^, ion exchange^8^,^9^ and so on. Compared with other traditional porous materials, such as zeolites and activated carbon compounds, crystalline microporous MOFs possess many outstanding features such as control of the functionalities in pores and unpredictably large surface areas^10^,^11^.

With the realization of open structures and permanent porosity in MOFs, though difficult to achieve, the immobilization of open metal sites (OMSs) and free functional organic sites (FOSs) on the pore surfaces has been regarded as effective strategy for the specific recognition and functional applications^12^,^13^. It has been generally assumed that the presence of OMSs will play a crucial role in Lewis acid catalysis^14^ and molecular recognition processes owing to their ability to impart highly selective and specific molecular transformations, transport, and storage^15^,^16^. Grafting FOSs such as -NH, -SH and -OH onto the surfaces of porous materials enhances adsorption of the acidic CO_2_ as well as heavy metal ions (HMI) by inducing different interactions with them^17^,^18^. However, such functionalization has the major drawback in practice of the easy interfere with the coordination chemistry because of the self-assembly of nodes with the functional organic sites. Among more than 10000 MOFs reported, only very few MOFs still possess FOSs after self-assembly without postsynthetic approaches^19^. Besides, inclusion of functional organic sites onto the pores surface also open up the postsynthetic approach for immobilization of novel metal ions precursors (Pd, Au, Ru, Pt, etc) for highly catalytic materials in organic syntheses and contaminant degradations^20^,^21^. The disadvantage of most MOFs reported containing functional organic sites are with the monodentate mode, which cannot act as strong supports for the metalation of metal nanoparticle^22^.

Designing MOFs with OMSs and FOSs mentioned above is meaningful and challenging for multi-functional applications. We have designed a flexible dicarboxylicacid ligand containing a functional amino-quinoxaline which possessing a chelating amino site (H_2_DDQ = *N*,*N*′-dibenzoicacid-2,3-diamino-quinoxaline)^23^. Two MOFs with H_2_DDQ have been synthesized previously with this ligand with the chelating amino sites connected to Zn(II) and Cu(II) ions unluckily^23^. When using the lanthanide ions (Y, Dy and Eu), three significative Ln-MOFs with free multisite FOSs have been constructed accompanied with rarely six-coordinated Ln ions accidentally. To our knowledge, six-coordinated Ln ions have never been reported in the structures of MOFs yet. All the frameworks are isostructural and show three-dimensional net of the **sra** topology. About 17.3 wt % (273 K) and 12.4 wt % (298 K) CO_2_ can be absorbed at and 760 Torr which suggest that the chelating amino groups and six-coordinated Ln ions have the cooperative interaction with the CO_2_ molecules. The porous Ln-MOFs also show anion-selective dyes adsorption and highly Lewis acidic catalysis for cyanosilylation reaction. After immobilizing Pd NPs on MOFs through graft interactions from free chelating amino groups using postsynthetic approach, the well dispersed Pd@Ln-MOF exhibited efficient and recyclable catalytic reduction of 4-nitrophenol to 4-aminophenol. Pd@Ln-MOF also acts as an efficient catalyst for Suzuki-Miyaura cross-coupling reactions with exposed Pd NPs.

## Methods

### Materials and Physical Measurements

All of the starting materials employed were purchased from commercial sources and used as received without further purification. Elemental analyses for C, H and N were determined with a Perkin-Elmer 240. Fourier transform infrared (FT-IR) spectra were measured as KBr pellets on a Nicolet FT-170SX spectrometer in the range of 400 cm^−1^–4000 cm^−1^. Raman spectra were measured as on a ThermoFisher DXR laser Raman spectrometer. Thermogravimetric analysis (TGA) experiments were carried out on an integrated thermal STA 449C analyzer heated from room temperature to 800 °C under N_2_ atmosphere. Powder X-ray diffraction (PXRD) patterns were collected on a Rigaku D/max2500VB3+/PC diffractometer equipped with Cu-Kα radiation (*λ* = 1.5406 Å). The morphology of Pd@Y-DDQ was investigated by High Resolution Transmission Electron Microscope JEM-2100 (HRTEM). The UV-Vis spectra were measured on UV-2450 spectrophotometer. ^1^H NMR spectra were recorded on Bruker AVANCE 500 MHz spectrometer at room temperature in CDCl_3_. ICP analysis was carried on Varian VISTA- MPX instrument. Details of the ligand synthesis were reported in the literature^23^.

### Synthesis of {[Y_3_(DDQ)_4_(H_2_O)_3_(NO_3_)]·7.08H_2_O·5.23DMF}_n_ (Y-DDQ)

A mixture containing H_2_DDQ (0.0300 g, 0.075 mmol) and Y(NO_3_)_3_·6H_2_O (0.0577 g, 0.15 mmol) in 2.5 mL of H_2_O/DMF (1:1) was sealed in a Teflon-lined autoclave and heated at 85 °C under autogenous pressure for four days and then allowed to cool to room temperature. The yellow crystals were washed with DMF, H_2_O and air-dried. Yield: 74% (based on H_2_DDQ). Anal. Calcd for C_103.68_H_104.7_ N_22.23_O_34.3_Y_3_ (*M*r: 2477.71): C, 50.21; H, 4.23; N, 12.56%. Found: C, 50.47; H, 4.12; N, 12.26%. IR (cm^−1^): 3362(w), 1656(m), 1604(s), 1565(w), 1522(s), 1486(m), 1390(s), 1308(m), 1242(m), 1179(w), 1153(w), 1091(w), 853(w), 780(s), 737(w), 679(w), 593(w), 568(w).

### Synthesis of {[Dy_3_(DDQ)_4_(H_2_O)_3_(NO_3_)]·5H_2_O·6DMF}_n_(Dy-DDQ)

The preparation of Dy-DDQ was similar to that of Y-DDQ except that Dy(NO_3_)_3_·6H_2_O (0.0686 g, 0.15 mmol) was used instead of Y(NO_3_)_3_·6H_2_O. The yellow crystals were washed with DMF, H_2_O and air-dried. Yield: 68% (based on H_2_DDQ). Anal. Calcd for C_106_H_114_N_23_O_33_Dy_3_ (*M*r: 2725.70): C, 46.71; H, 4.22; N, 11.82%. Found: C, 46.41; H, 4.39; N, 11.54%. IR (cm^−1^): 3360(w), 1655(m), 1606(s), 1564(w), 1522(s), 1488(m), 1391(s), 1308(m), 1245(m), 1176(w), 1155(w), 1093(w), 850(w), 781(s), 739(w), 677(w), 594(w), 566(w).

### Synthesis of {[Eu_3_(DDQ)_4_(H_2_O)_3_(NO_3_)]·5H_2_O·6DMF}_n_(Eu-DDQ)

The preparation of Eu-DDQ was similar to that of Y-DDQ except that Eu(NO_3_)_3_·6H_2_O (0.0612 g, 0.15 mmol) was used instead of Y(NO_3_)_3_·6H_2_O. The yellow crystals were washed with DMF, water and air-dried. Yield: 55% (based on H_2_DDQ). Anal. Calcd for C_106_H_114_N_23_O_33_Eu_3_ (*M*r: 2694.08): C, 47.26; H, 4.27; N, 11.96%. Found: C, 46.97; H, 4.32; N, 11.77%. IR (cm^−1^): 3358(w), 1654(m), 1605(s), 1567(w), 1526(s), 1490(m), 1389(s), 1306(m), 1247(m), 1179(w), 1152(w), 1094(w), 853(w), 779 (s), 741(w), 675(w), 596(w), 569(w).

### Synthesis of Pd@Y-DDQ

Pd(OAc)_2_ (28 mg, 0.11 mmol) was dissolved in 3 mL acetone, yellow Y-DDQ crystals (46 mg, 0.05 mmol) was placed into the Pd-based solution for 24 hours quietly at room temperature. The solid was obtained after centrifugation and washed with acetone (3 × 10 mL) and then slowly dried under vacuum at 50 °C for 8 h to obtain brown Pd(II)@Y-DDQ crystals. 100 mg activated Pd(II)@Y-DDQ were thermally reduced in a hydrogen stream at 160 °C for 4 h. The Pd loading on the sample was 1.2 wt% based on ICP analysis.

### X-ray Crystallography

The X-ray intensity data for the three compounds were collected on a Rigaku Saturn 724+ CCD diffractometer with graphite monochromatized Mo K*α* radiation (*λ* = 0.71073 Å). The crystal structures were solved by direct methods using difference Fourier synthesis with SHELXTS^24^, and refined by full-matrix least-squares method using the SHELXL-97 program^25^. The non-hydrogen atoms were refined with anisotropic displacement parameters. Hydrogen atoms except for those of guest molecules were added according to theoretical models. To Dy-DDQ and Eu-DDQ, the solvent molecules and anion part of the structures were highly disordered and impossible to been found in the Fourier maps and fixed in the ideal position^26^. To resolve this issue, the contribution of solvent and anionic electron density was removed by SQUEEZE routine in PLATON^27^. The molecules removed were determined with elemental analysis and TG data. Crystal data and details of the structure determination for the three compounds are listed in Table S1.

### Gas Adsorption Measurements

Prior to gas adsorption experiments, the samples were soaked in methanol to exchange H_2_O and DMF solvents, which was then followed by evacuation under a dynamic vacuum at 120 °C for the 8 hours. All the gas adsorption isotherms were measured using a Micromeritics ASAP 3Flex analyzer employing a standard volumetric technique up to saturated pressure. The N_2_ and Ar adsorption isotherms were monitored at 77 K and 87 K respectively, while CO_2_ adsorption isotherms were obtained at 273 K using a dry ice-acetone bath. The adsorption data were refitted to the Brunauer-Emmett-Teller (BET) equation to determine the BET surface area.

### Dye Adsorption Experiments

Compound (5 mg) was added into a 40 mL of 50 mg/L dye-containing water solution under stirring at room temperature. The solution was centrifuged, and the clear liquid was analyzed by UV-vis absorption spectroscopy.

### Catalytic Test for Aldehyde and Ketone Cyanosylation Reaction

Into a 10 mL screw-cap vial was successively placed aldehyde (1.0 mmol) in trimethylsilyl cyanide (TMSCN, 2 mmol) and desolvated compound (2.5 mol %) was then added to initiate the reaction with ultrasound for an hour in the sealed vial. To less reactive the ketone cyanosylation reaction, the reaction time extended to two hours with the same ratio of the reactants. After the reaction completed, the catalyst was removed by centrifugation and then filtered with ethyl acetate quickly. The conversion of aldehydes and ketones were determined by gas chromatography (GC, Agilent 7890A) analysis and GC-MS (HP 6890) spectra with those of authentic samples.

### Catalytic reaction of reduction of 4-nitrophenol to 4-aminophenol

Typically, 180 mg of NaBH_4_ was dissolved in 10 mL deionized water and then mixed with 20 mL of 7 mg 4-nitrophenol. The mixture was stirred for 2 min, and then 5 mg of catalyst was added. After introducing the catalyst, the bright yellow solution turned to be clear gradually. UV-vis spectra of the solution were measured during the course of the reaction.

### Activity Test for Suzuki-Miyaura Coupling Reaction

Phenylboronic (0.15 mmol), aryl halide (0.1 mmol), base (0.2 mmol), and catalyst (5 mg) were stirred vigorously in deionized water (2 mL) at 90 °C for 5 h under mild stirring. The yields of reaction products were analyzed by GC (Agilent 7890A). All the productions were separated and the spectra of ^1^HNMR were provided in supporting information.

## Results and Discussion

### Synthetic and Spectral Aspects

#### Structure description

**Figure 1:**
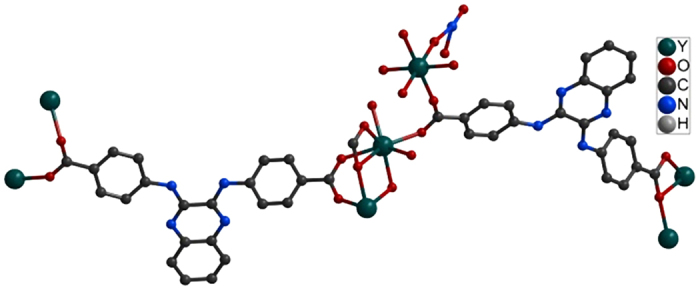
View of the asymmetric unit of Y-DDQ.

**Figure 2:**
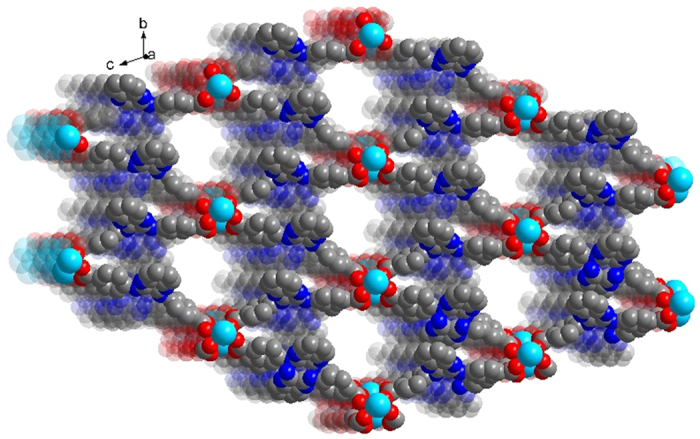
An infinite 3D framework with 13.798 × 12.422 Å dimensions along the a-axis.

**Figure 3:**
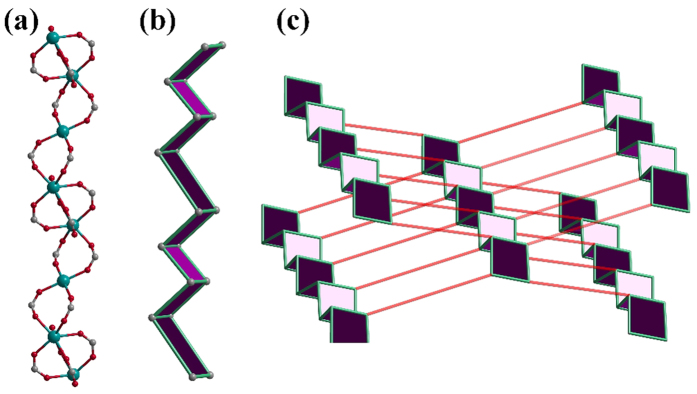
(**a**) 1D metal chain. (**b**) A zigzag ladder simplified from the metal chain. (**c**) A simplified three-dimensional net of the **sra** topology of Y-DDQ.

All the MOFs are isostructural, the structures and properties of Y-DDQ are described in detail here as a representative example. Y-DDQ crystallizes in the triclinic space group *P*ī, The ORTEP view of Y-DDQ is shown in Fig. 1. One kind of Y center adopts a bicapped trigonal-prismatic geometry with seven oxygen donors from carboxylate groups and one from coordinated water molecules occupying eight coordination sites. Interestingly, the other kind of Y atoms is six-coordinated which has never been found in MOFs as we know. The six-coordinated Ln^3+^ ions are formed due to the strong hindrance. The six-coordinated Ln^3+^ ions exist between the two eight-coordinated Ln^3+^ ions with the distance of 4.811 Å. The coordination environments of the two eight-coordinated Ln^3+^ ions made the space of the central six-coordinated Ln^3+^ ions so crowded. There are eight benzoic acidic groups of H_2_DDQ ligands around the six-coordinated Ln^3+^ ions, making other coordinated groups hardly link to Ln^3+^ ions. All these make strong steric hindrance to the six-coordinated Ln^3+^ ions. The two carboxylate groups of DDQ^2−^ exhibit two different coordination modes: *μ*_2_-*η*^1^: *η*^1^ and *μ*_2_-*η*^2^: *η*^1^ modes chelating the Y atoms. Adjacent pairs of Y centers are bridged by carboxylates to form one-dimensional metal chains along *c* axis. Each 1D metal chain is connected through DDQ^2−^ to extend into an infinite 3D framework with 13.798 Å × 12.422 Å dimensions (atom-to-atom distance) along *a* axis (Fig. 2), where DMF and H_2_O molecules are located. From the topological point of view, the SBU consists of infinite (-O-Y-)_∞_ rods, while the carboxylate C atoms are at the vertices of a zigzag ladder SBU. A three-dimensional net of the **sra** topology is constructed by H_2_DDQ linkers joining the zigzag ladders (Fig. 3). As we know, MIL-47, MIL-53 and MOF-71 are all **sra** topology consisting of similar rods with different metal centers^28^. PLATON calculated suggested a solvent-accessible volume of 1159.8 Å^3^ (approximately 40.7% of unit cell) by excluding the guest H_2_O and DMF molecules. The pore surface decorated with functional amino- quinoline decreases obviously compared to other MOFs of **sra** topology reported.

The three MOFs were characterized by PXRD, TGA analysis and FT-IR spectra. All the experimental PXRD patterns were measured at room temperature (Figure S2). The peak positions of the simulated and experimental PXRD patterns are in agreement with each other, suggesting the good phase purity of the three MOFs. PXRD pattern of activated Y-DDQ indicates that the framework is stable after removing the solvents in the channels (Figure S3). The chemical stability of Y-DDQ was also examined by suspending samples in different solvents for one week. The PXRD patterns collected for each samples confirmed that Y-DDQ retaining their crystalline after immersing in different solvents (Figure S4). TG analysis of the three MOFs revealed that they have good thermal stabilities, since the framework start to decompose beyond 400 °C (Figure S5).

**Figure 4:**
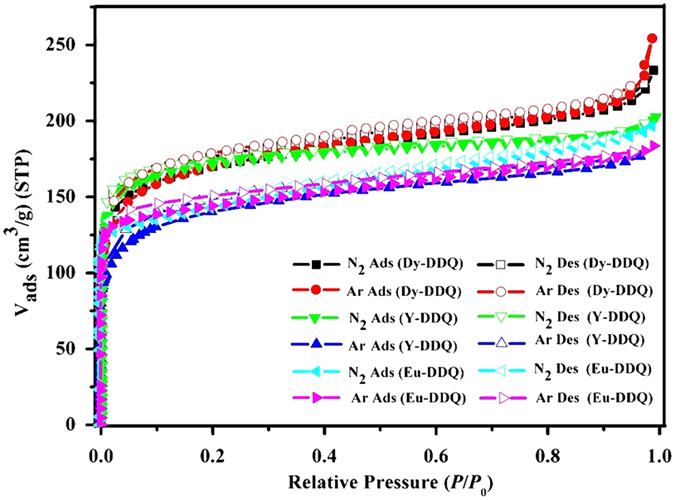
N_2_ and Ar adsorption isotherms for Y-DDQ, Dy-DDQ and Eu-DDQ.

**Figure 5:**
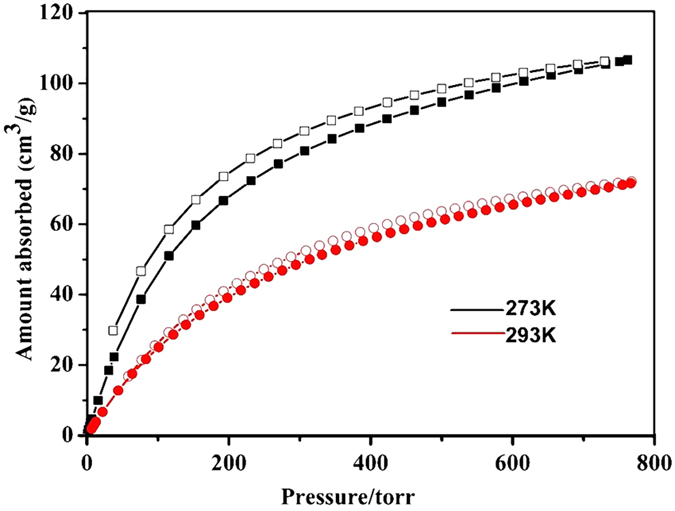
CO_2_ adsorption isotherms in Y-DDQ at 273 and 298 K.

For gas adsorption properties, activated Ln-DDQs were selected to study the permanent porosities. As shown in Fig. 4, N_2_ and Ar adsorption isotherms of Ln-DDQs both reveal typical type-I behaviors, indicating the microporous structures. To Y-DDQ, the uptakes of N_2_ and Ar increase dramatically when pressurized and reach to about 202.4 cm^3^ g^−1^ (STP) and 176.9 cm^3^ g^−1^ (STP) respectively. N_2_ adsorption indicates a surface area of 658.8 m^2^ g^−1^ using the standard Brunauer-Emmett-Teller (BET) model. In addition, the surface area is calculated to be 525.9 m^2^ g^−1^ according to Ar adsorption. The relatively higher uptakes of N_2_ and Ar are 233.2 cm^3^ g^−1^ (STP) and 254.4 cm^3^ g^−1^ (STP) with the surface areas are 634.1 m^2^ g^−1^ (N_2_) and 635.9 m^2^ g^−1^ (Ar) for Dy-DDQ. The surfaces areas are of Eu-DDQ are 545.4 m^2^ g^−1^ (N_2_) and 562.3 m^2^ g^−1^ (Ar) according to the N_2_ and Ar adorption isotherms. Using the Horvath-Kawazoe (HK) method, the pore sizes of Ln-DDQs distribution from the N_2_ and Ar adsorption isotherms are about 7.2 Å (Y-DDQ), 5.1 Å (Dy-DDQ) and 5.0 Å (Eu-DDQ) (Figures S7–S9). From the similar the porous properties of the three MOFs, we can make some reasonable inferences about the similar adsorption capacities of all Ln-DDQs. BET surface areas of Ln-DDQs are smaller than MIL-47 and MIL-53, though with the similar topology and larger organic linker^29^,^30^. The quinoline rings in the ligand should be the leading cause of surface area decrease. Taking the free chelating amino groups as well as the six-coordinated Ln(III) ions into consideration, the CO_2_ adsorption of Y-DDQ was investigated as a representative example. It was observed that Y-DDQ reversibly adsorbs a significant amount of CO_2_ (106.7 cm^−3^ g^−1^, 17.3 wt %) at 273 K and 760 Torr (Fig. 5), which is higher than that of MIL-53 (ca. 60 cm^−3^ g^−1^)^31^,^32^. While at 298 K, MIL-53 and MIL-47 can uptake about 10.6 and 8.1 wt % respectively^31^. However, the CO_2_ uptake of Y-DDQ at 298 K is higher than that of MIL-53 and MIL-47 of about 12.4 wt %. In contrast to most MOFs reported, the CO_2_ adsorption of Y-DDQ is comparable in low pressure in the absence of any postsynthetic modification^31^.

**Figure 6:**
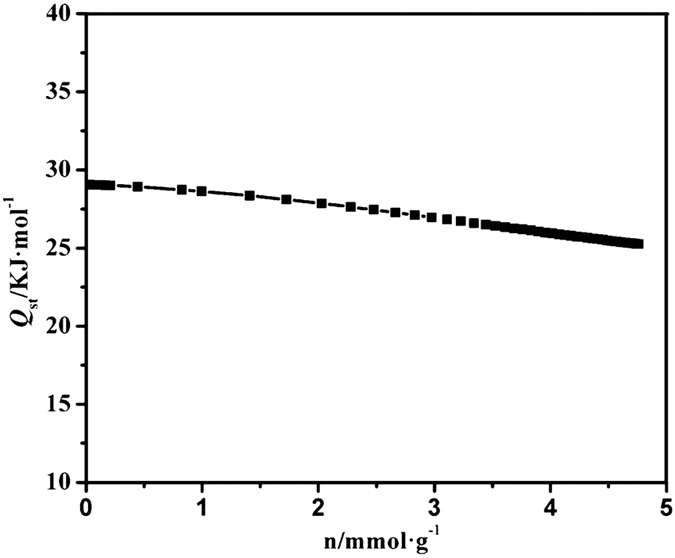
Isosteric heats of CO_2_ adsorption (Qst) value for Y-DDQ calculated using isotherms collected at 273 and 298 K.

The isosteric heat (*Q*_st_) of CO_2_ was obtained on the basis of two isotherms at 273 and 298 K (Fig. 6), followed by a fit of the data to virial equation^32^. The first preferential adsorption site, Δ*H*_ads_ = 29.3 KJ mol^−1^, can be attributed to the evident interactions between CO_2_ molecules and both free chelating amino sites and six-coordinated Y(III) ions. After the first loading, Δ*H*_ads_ falls very slowly to about 25.3 KJ mol^−1^, indicating the relatively weaker CO_2_ interactions at higher loading. As a result, the introduction of the chelating amino groups and OMSs can significantly enhance the CO_2_ uptake due to the cooperative interactions between CO_2_ molecules and the active sites.

**Figure 7:**
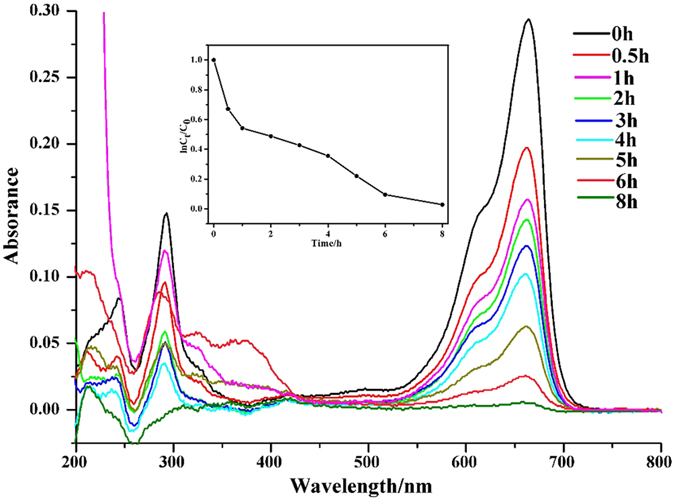
UV-vis absorption spectra of MB solution and the relationship between *C*_t_/*C*_0_ and reaction time (*t*) in the absorption of MB with Y-DDQ.

**Figure 8:**
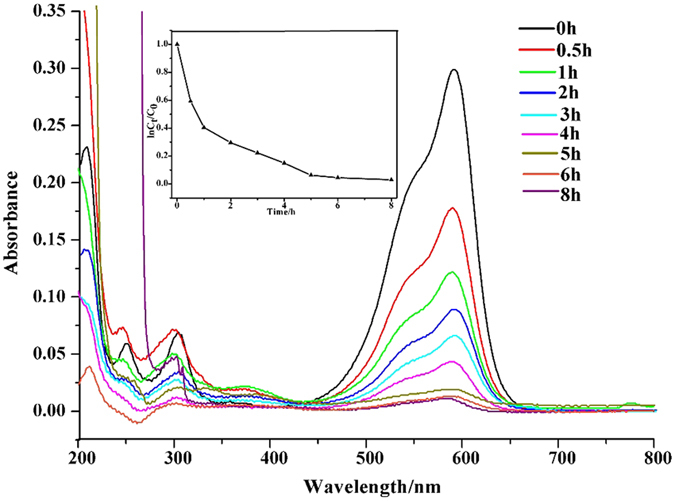
UV-vis absorption spectra of CV solution and the relationship between *C*_t_/*C*_0_ and reaction time (*t*) in the absorption of CV with Y-DDQ.

Recently, MOFs with higher porosity and active sites have been received increasing attention for liquid adsorption on removal of dyes from the aqueous solutions^33^,^34^. When Y-DDQ were added into water solutions of three harmful dyes [methyl blue (MB), crystal violet (CV) and methyl orange (MO)] at room temperature for 8 h, the dyes-loading abilities were measured by UV-vis absorption spectroscope (Figs 7 and 8 and S12). The dye uptakes of Y-DDQ were about 401 mg/g^−1^ for MB, 308 mg/g^−1^ for CV and 130 mg g^−1^ for MO. The pore size of Y-DDQ is large enough for all the three dyes to fill in due to the larger pore size and possible breathing transitions after activated of **sra** topologies^35^. The three dyes selected can be divided into two types, one is neutral dye (MO) and the others are ionic dyes (MB and CV). The most possible reason for ionic dyes with higher uptakes is the six-coordinated Y(III) ions providing open sites for the Cl^−^ anions to coordinate. The channels possess negative charges after Cl^−^ anions connecting to the Y(III) ions, and the cationic part of dyes will have high affinity in the channels. As we know, the mechanism of most MOFs reported for dyes adsorptions are the result of charge balance, the reactions between the dyes and FOSs or pores with large sizes^36^,^37^. Using open metal sites for enhancement of the ionic dyes adsorption has never been reported in MOFs up to now. It will provide a new conception for dye-containing wastewater treatment with MOFs.

Ln-MOFs have been widely used to catalyze cyanosilylation reaction due to the high Lewis acid and flexible coordination modes^38^,^39^. As the work we reported^23^, this reaction presents higher catalytic activities in the absence of solvents at room temperature (Figure S18). Y-DDQ showed more than 99% conversion in the cyanosilylation of benzaldehyde and 4-chlorobenzaldehyde in only one hour with a TOF of 39.6 h^−1^ (Table 1). Though extending reaction times to two hours for catalyzing ketones, the yields of acetophenone and 2-chloroacetophenone were less than 30%. However, 4-chloroacetophenone and 4-bromo-acetophenone afforded higher yields (79% and 87%). The heterogeneity of the reaction was confirmed by the filtration test (Table S3). Successive reactions were carried out for cyanosilylation of benzaldehyde, indicating that the recovered catalyst can be reused without an appreciable loss after five cycles (Figure S14). Such comparable catalytic behavior^40^ is attributed to strong Lewis acidity of Y-DDQ with six-coordinated Y(III) centers which provide open metal sites for this catalytic system.

**Figure 9:**
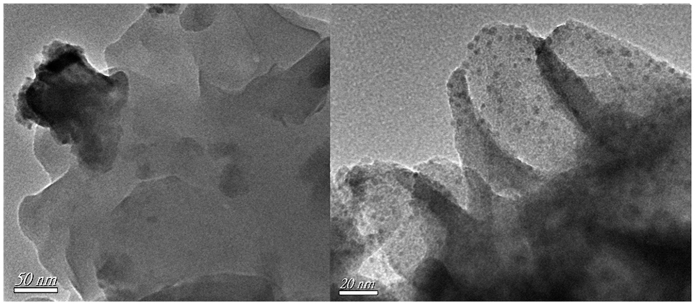
(Left) HRTEM images of Y-DDQ. (Right) HRTEM images of Pd@Y-DDQ.

Recently, MNPs have been widely explored for enhanced catalytic sites due to the active catalytic centers on the surface of MNPs. In view of the chelating amino groups as a functional sites for postsynthesize, 1.2 wt% Pd NPs were prepared on Y-DDQ as support (called as Pd@Y-DDQ) by strong interactions with chelating amino groups. PXRD patterns of Pd@Y-DDQ did not show apparent transformation of crystallinity (Figure S17). Two theta degrees of Pd at about 40° cannot see very clearly because of the relatively smaller Pd-loading amount^41^. High-resolution TEM images of Pd@Y-DDQ give evidence for embedded Pd NPs with size distributions of 2.5–4 nm (Fig. 9). The Pd NPs are well dispersed on the surface of Y-DDQ and no apparent aggregation on the surface of Y-DDQ can be seen. CO_2_ adsorption of Pd@Y-DDQ was also investigated in Figure S11. Part of chelating amino groups occupied by the Pd NPs leads to nearly half decrease of CO_2_ adsorption of Pd@Y-DDQ compared to Y-DDQ. The Pd_3d3/2_ and Pd_3d5/2_ XPS spectrum of Pd@Y-DDQ is shown in Figure S10. The signals at 340.6 eV (3d3/2) and 335.2 eV (3d5/2) are characteristic of Pd metal^42^.

**Figure 10:**
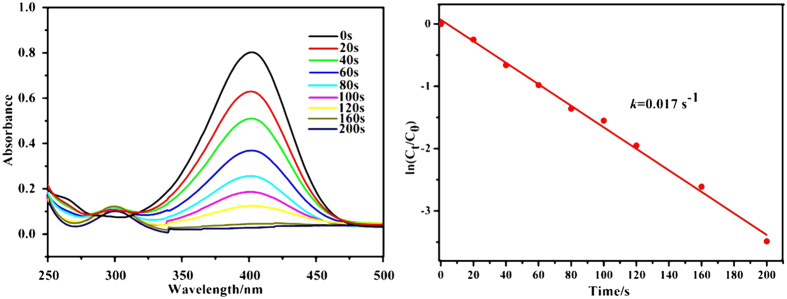
(Left) UV-vis spectra of 4-nitrophenol reduction. (Right) Effect of Pd@Y-DDQ on the reduction rate of 4-NP.

The capability of catalytic performance like liquid-phase reduction of 4-nitrophenol to 4-aminophenol by NaBH_4_ is the most important function for metal Nanoparticles to monitor the catalytic reaction kinetics. This rapid reaction can be measured using UV-vis spectroscope. As we know, 4-nitrophenol cannot be reduced by aqueous NaBH_4_ in the absence of metal NPs^43^. When Pd@Y-DDQ catalyst was introduced into the solution containing NaBH_4_ and 4-nitrophenol, the absorption peak at 400 nm of 4-nitrophenol decreased quickly along with an increase of the 300 nm peak of 4-aminophenol concomitantly (Fig. 10). The complete reduction of 4-nitrophenol was achieved within 200 s over the Pd@Y-DDQ catalyst. The overall kinetic analysis of the 4-nitrophenol reduction reaction is presented as ln(*C*_t_/*C*_0_) = −*k*t, where *k* is the kinetic rate constant, *C*_0_ and *C*_t_ are the initial and apparent concentrations of 4-nitrophenol, respectively^44^. The ln(*C*_t_/*C*_0_) is plotted as a function of time, where the slope of the best fit line represents the −*k* value of the reaction. A linear relationship between ln(*C*_t_/*C*_0_) and time (*t*) was observed for Pd@Y-DDQ catalyst (Fig. 10), indicating that this catalytic reduction reaction can be considered as a pseudo-first order reaction with the *k* = 0.017 s^−1^. The reaction rate constant obtained with the Pd@Y-DDQ catalyst is comparable to most Pd based catalysts reported^45^,^46^. The probable mechanism of high catalytic activity may be explained as follows: 4-nitrophenol can be adsorbed onto Pd@Y-DDQ via supramolecular interactions between 4-nitrophenol and the framework of Y-DDQ. Because of the OMSs in the structure, coordination effects between the hydroxyl group of 4-nitrophenol and six-coordinated Y(III) ions can also fix 4-nitrophenol onto the support well. Then the electron from 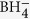 transfers to Pd NPs and subsequently the 4-nitrophenol absorbed on the catalyst surface takes electrons and transforms to 4-aminophenol^44^. What makes the comparable conversion is the high concentration of 4-nitrophenol on the support with the help of OMSs, which can enhance the catalytic reduction of 4-nitrophenol via more chance to react with the Pd NPs. The catalyst did not show apparent loss of the activities after eight cycles, indicating the strong adhesion force on the MOF support with Pd NPs (Figure S16).


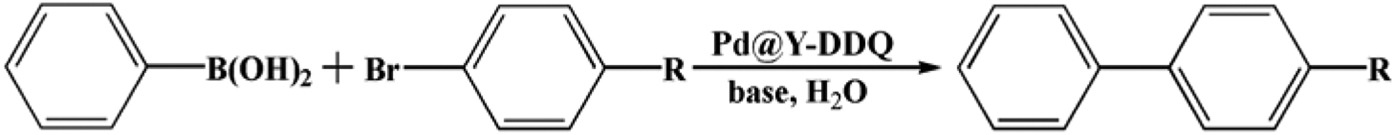


**Figure 11:**
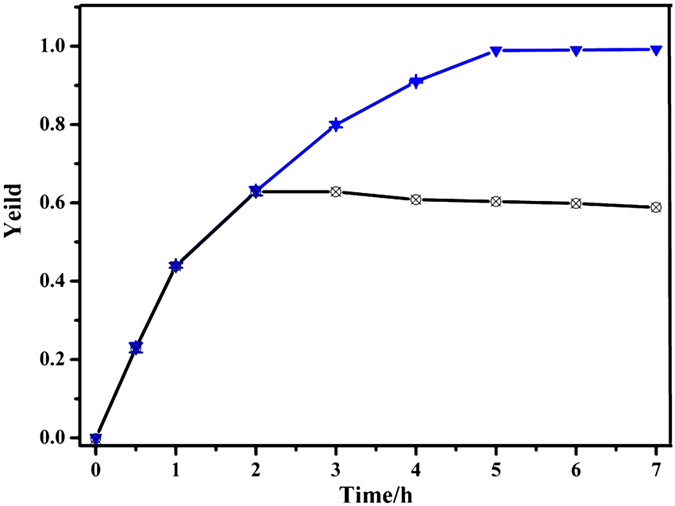
Kinetic profile for Suzuki-Miyaura coupling reactions reaction catalyzed by Pd@Y-DDQ, removal of Pd@Y-DDQ after 2 h.

C-C bond formation reactions are of great importance in organic synthesis. Suzuki-Miyaura cross-coupling reaction of aryl halides with phenylboronic acids is one which has been widely used to evaluate the catalytic performance with Pd compounds. The effect to the catalytic property was firstly investigated on bromobenzene with phenylboronic acid using different base in deionized water at 90 °C for 5 h in the presence of Pd@Y-DDQ. All the bases selected affected the yields well, especially with the highest yields when using Cs_2_CO_3_ and NaOH as the base (Table 2). As a result, Cs_2_CO_3_, which is a weaker base than NaOH, was chose to test other substrates with different groups. We compared the activities of phenylboronic acid with *p*-bromotoluene, *p*-bromoacetophenones, *p*-bromo-benzonitrile. *p*-bromoacetophenones reached nearly full conversion within 5 hours, while *p*-bromotoluene and *p*-bromobenzonitrile were converted into the corresponding products in 92% and 98% yields respectively. The presence of -CH_3_ group on the bromobenzene slightly suppresses the reaction activity, which is due to electron-donating substitutes stabilizing the C-halides bond against activation^47^. On the other hand, the yields are almost 100% with -COCH_3_ and -CN groups because of the electron-withdrawing effect. To make a comparison, Pd(OAc)_2_ and Pd(II)@Y-DDQ were also used as catalysts for this reaction. As predicted, the product yields were much lower than Pd@Y-DDQ. The conversions stop increasing immediately after removal of Pd@Y-DDQ, indicating the heterogeneity of this catalytic reaction (Fig. 11). In comparison with Pd/C materials^48^,^49^ (entry 17 and 18), Pd@Y-DDQ required less catalyst-loading and showed higher reaction yeilds. The high catalytic activity of Suzuki-Miyaura cross-coupling reaction should be attributed to the exposed and well-dispersed Pd NPs on the MOF support. Pd@MIL-101Cr-NH_2_^50^ and Pd/MIL-53(Al)-NH_2_^51^ reported (entry 19 and 20) also showed excellent catalytic activities. This enhancement may be attributed to the presence of free amino groups in the framework which make the well-dispersed Pd NPs on MOF without aggregation during the process of metalation through strong coordination with Pd(OAc)_2_.

## Discussion

In conclusion, three new synthesized Ln-DDQs were constructed containing free chelating amino groups as FOSs and a rare example of six-coordinated Ln(III) ions as OMSs. To our knowledge, Ln-DDQs are the first example for a framework with both free chelating N-H groups and six-coordinated Ln(III) ions together. Owing to presence of OMSs and FOSs, Ln-DDQs show good abilities of CO_2_ and dyes captures and Lewis acid catalysis. Using postsynthetic approaches embedding Pd NPs on Y-DDQ, well-dispersed Pd@Y-DDQ showed high catalytic activities for reduction of 4-nitrophenol and the Suzuki–Miyaura cross-coupling reaction. Using free chelating amino groups to embed metal NPs in MOFs here has been proved to be an effective strategy for pursuing high active catalyst. Finally, these Ln-DDQs provide a new approach for MOFs with high applied performances in the families of MOFs.

## Additional Information

**How to cite this article**: Zhu, Y. *et al*. Lanthanide Metal-Organic Frameworks with Six-Coordinated Ln(III) Ions and Free Functional Organic Sites for Adsorptions and Extensive Catalytic Activities. *Sci. Rep.* **6**, 29728; doi: 10.1038/srep29728 (2016).

## Supplementary Material

Supplementary Information

## Figures and Tables

**Table 1:**
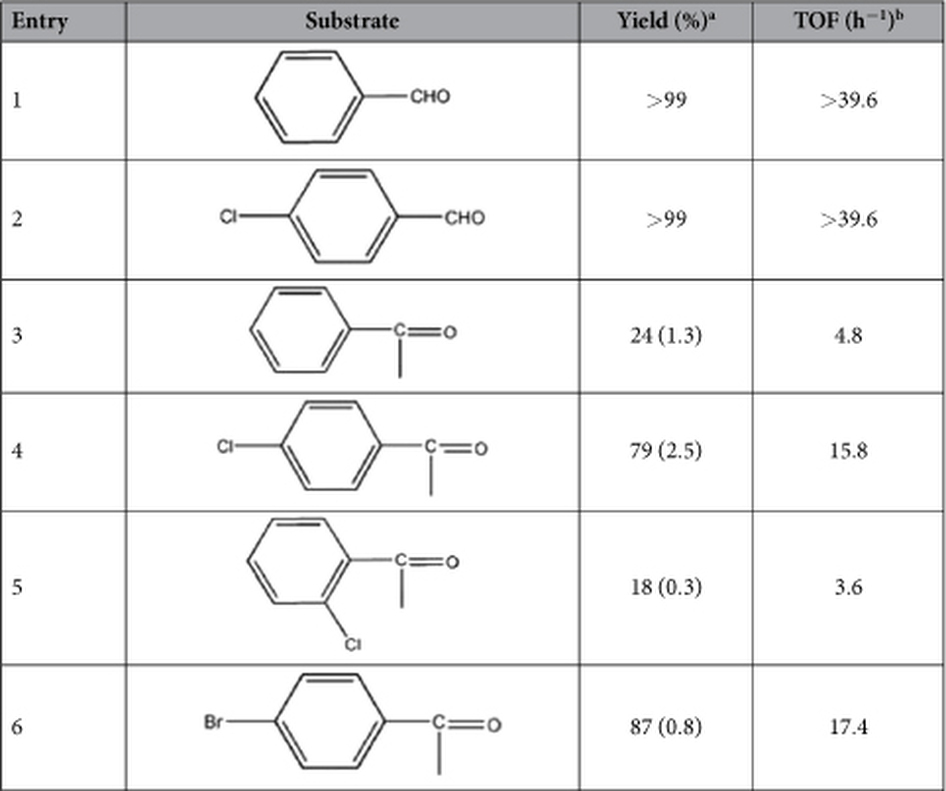
Comparison of the catalytic activity for Y-DDQ: aldehydes or ketones cyanosilylation reaction performed with different substrates.

**Table 2:** Suzuki–Miyaura coupling reactions catalyzed by Pd@Y-DDQ.

Entry	R	Base	T/h	Yield/%^c^
1	H	NaOH	5	99 (0.4)
2	H	Na_2_CO_3_	5	95 (0.3)
3	H	K_2_CO_3_	5	97 (0.8)
4	H	Cs_2_CO_3_	0.5	23 (1.2)
5	H	Cs_2_CO_3_	1	44 (0.6)
6	H	Cs_2_CO_3_	2	63 (1.1)
7	H	Cs_2_CO_3_	3	80 (0.7)
8	H	Cs_2_CO_3_	4	91 (0.3)
9	H	Cs_2_CO_3_	5	>99
10	H	Cs_2_CO_3_	6	>99
11	H	Cs_2_CO_3_	7	>99
12	CH_3_	Cs_2_CO_3_	5	92 (0.7)
13	COCH_3_	Cs_2_CO_3_	5	>99
14	CN	Cs_2_CO_3_	5	98 (0.6)
15	H	Cs_2_CO_3_	5	48 (0.4)^d^
16	H	Cs_2_CO_3_	5	55 (0.6)^e^
17	H	Cs_2_CO_3_	5	92 (0.9)^f^
18	CH_3_	NaOH	6	96^g^
19	CN	K_2_CO_3_	0.67	trace^h^
20	CH_3_	Cs_2_CO_3_	6	>99^i^
21	COCH_3_	Na_2_CO_3_	0.5	94^j^

^c^Isolated yields were determined by GC analysis for three runs averagely (the value in the parentheses is error of the mean).

^d^Catalyzed by Pd(OAc)_2_.

^e^Catalyzed by Pd(II)@Y-DDQ.

^f^Result from 5 wt% Pd/C purchased from Xiya.

^g^Result from ref. 48 (5 wt% Pd/C).

^h^Result from ref. 49 (5 wt% Pd/C).

^i^Result from ref. 50 (8 wt% Pd@MIL-101Cr-NH_2_).

^j^Result from ref. 51 (0.19 wt% Pd/MIL-53(Al)-NH_2_).
