# Supplementary material for: Lanthanide Metal-Organic Frameworks with Six-Coordinated Ln(III) Ions and Free Functional Organic Sites for Adsorptions and Extensive Catalytic Activities
Source: Sci Rep. 2016 Jul 19;6:29728. doi: 10.1038/srep29728 (PMC4949474; doi:10.1038/srep29728)

Supporting Information

Lanthanide Metal-Organic Frameworks with Six-Coordinated Ln(III) Ions and Free Functional Organic Sites for Adsorptions and Extensive Catalytic Activities

Yu Zhu[1], Min Zhu[2], Li Xia[3], Yunlong Wu[3], Hui Hua[1], Jimin Xie[3]

[1]College of Pharmacy and Chemistry & Chemical Engineering,Taizhou University, Taizhou, 225300, China

[2]Hanlin College, Nanjing University of Chinese Medicine, Taizhou, 225300, China

[3]School of Chemistry and Chemical Engineering, Jiangsu University, Zhenjiang, 212013, China

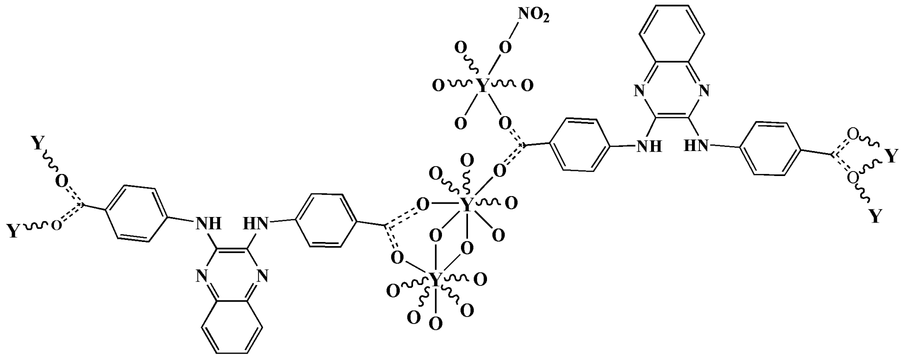

Figure S1. Coordination diagram of Y-DDQ

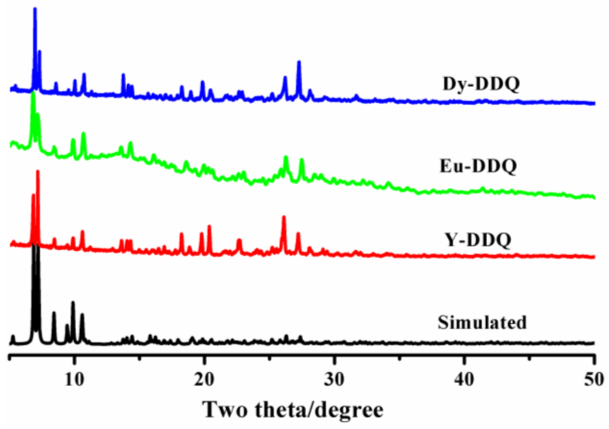

Figure S2. PXRD patterns of Y-DDQ, Dy-DDQ and Eu-DDQ.

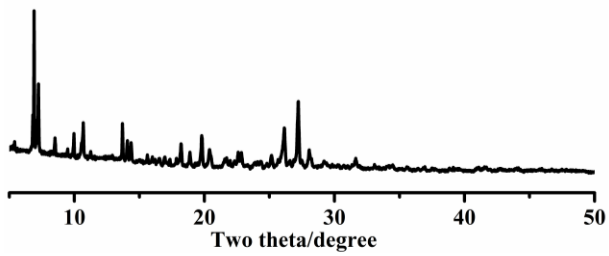

Figure S3. PXRD patterns of activated Y-DDQ.

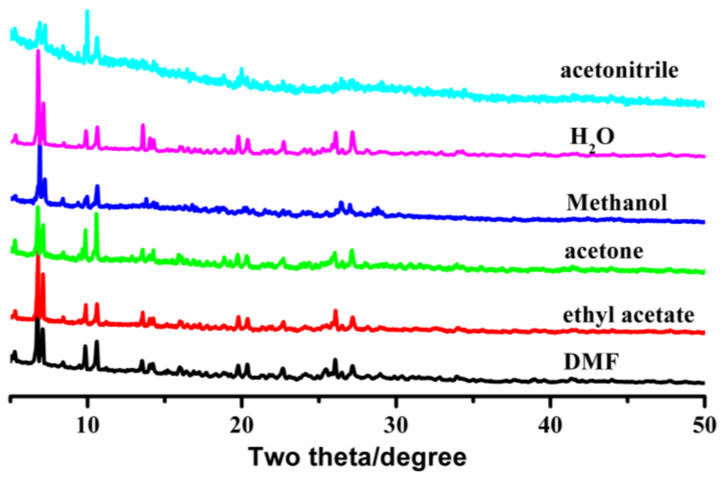

Figure S4. PXRD patterns of Y-DDQ in different solvents for one week.

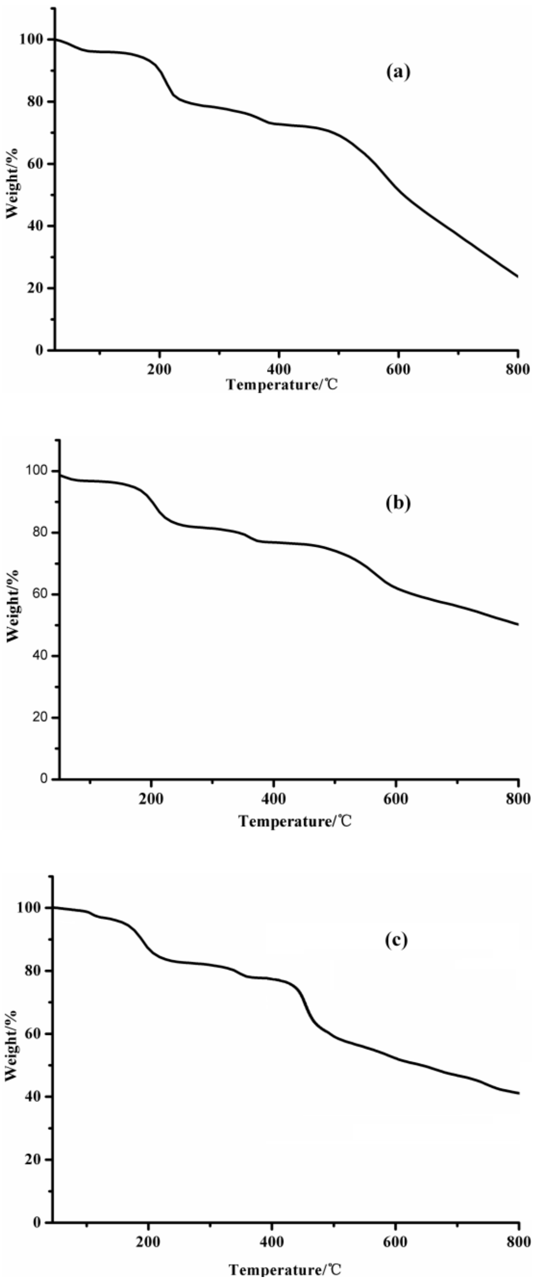

Figure S5. TG profiles of Y-DDQ (a), Dy-DDQ (b) and Eu-DDQ (c).

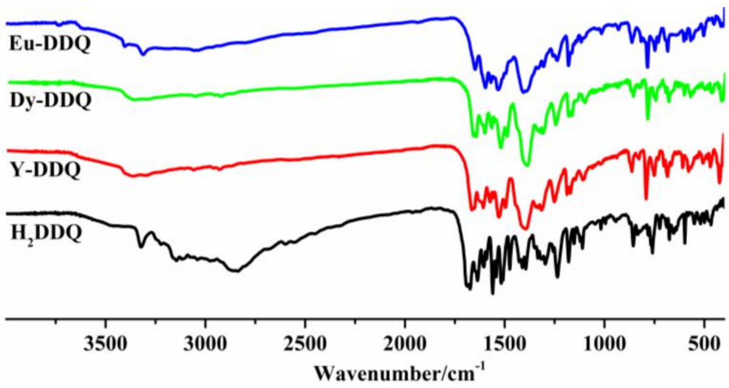

Figure S6. IR spectra of H_2DDQ, Y-DDQ, Dy-DDQ and Eu-DDQ.

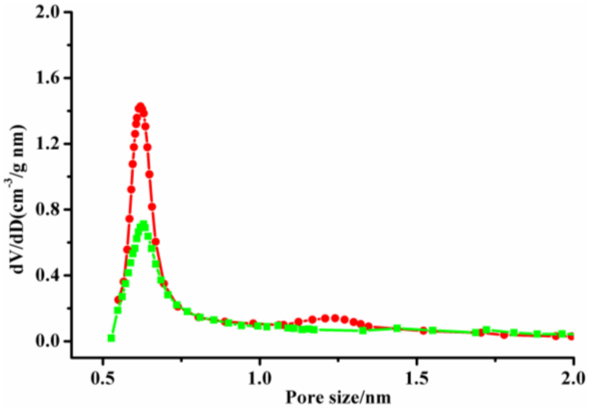

Figure S7. Pore size distributions for Y-DDQ from N_2 (red) and Ar (green) adsorption isotherms.

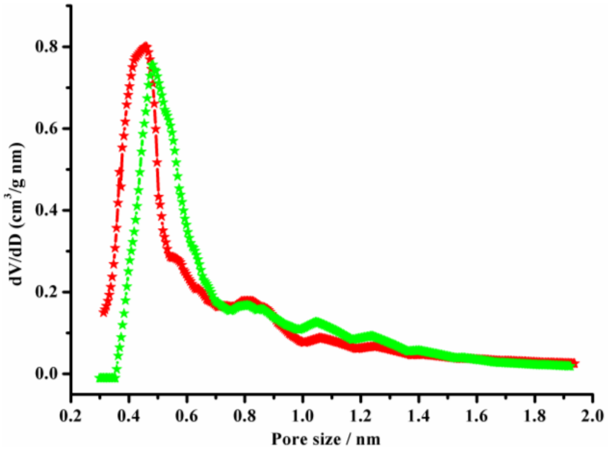

Figure S8. Pore size distributions for Dy-DDQ from N_2 (red) and Ar (green) adsorption isotherms.

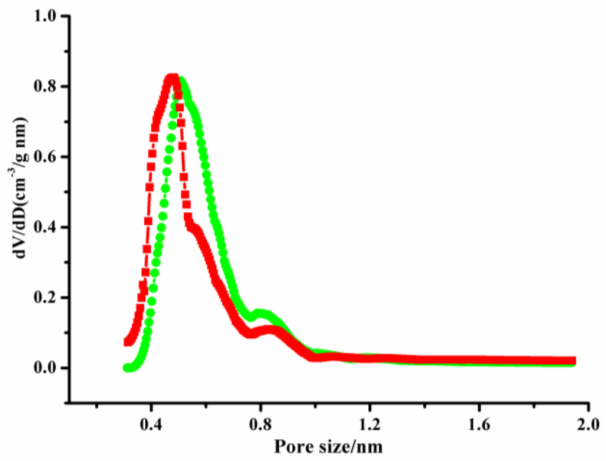

Figure S9. Pore size distributions for Eu-DDQ from N_2 (red) and Ar (green) adsorption isotherms.

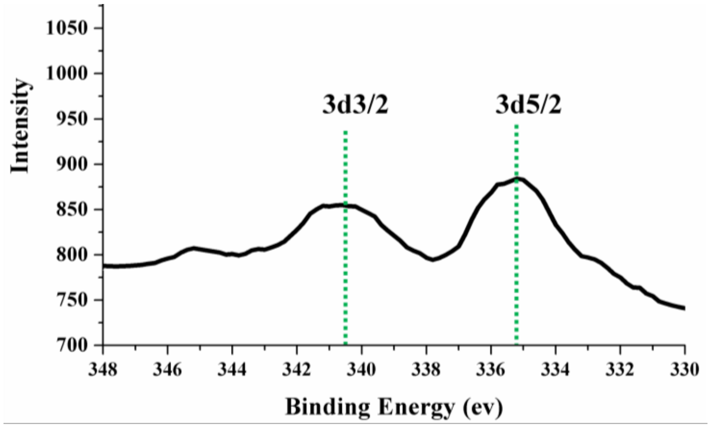

Figure S10. The XPS analysis of Pd NPs.

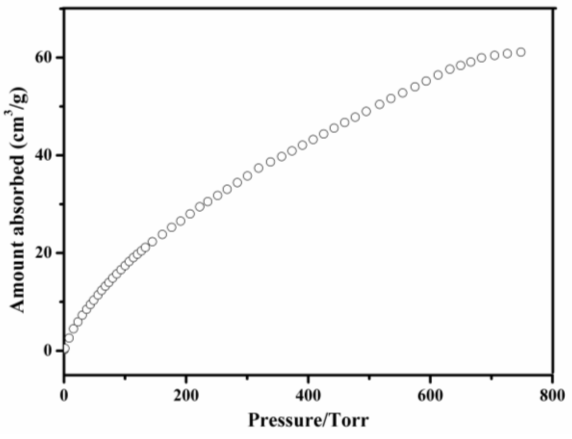

Figure S11. CO_2 adsorption of Pd@Y-DDQ at 273 K.

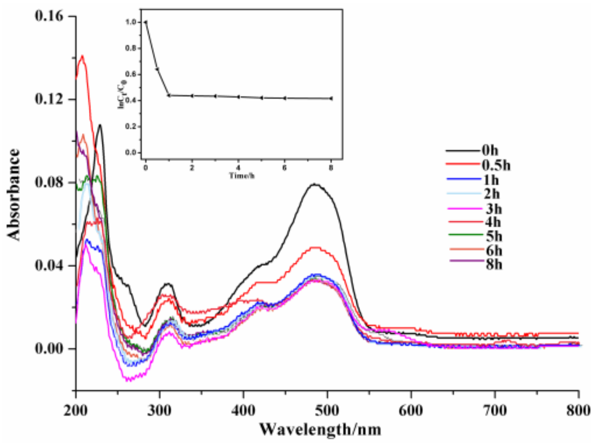

Figure S12. UV-vis absorption spectra of MO solution and the relationship between C_t/C_0 and reaction time (t) in the absorption of MO in the presence of Y-DDQ

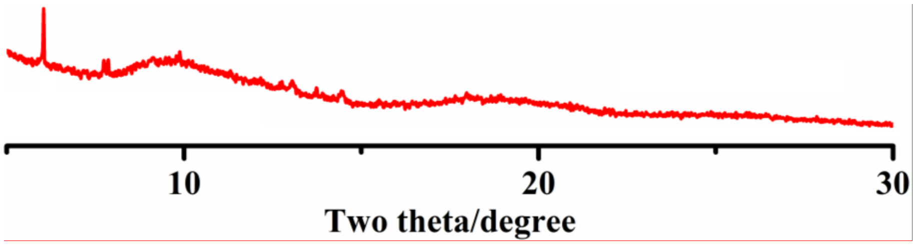

Figure S13. PXRD pattern of Y-DDQ after catalyzing cyanosilylation of benzaldehyde

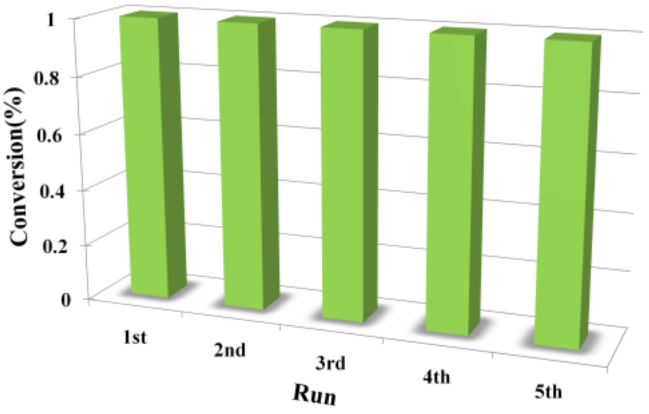

Figure S14. Recycling tests for cyanosilylation of benzaldehyde catalyzed by Y-DDQ.

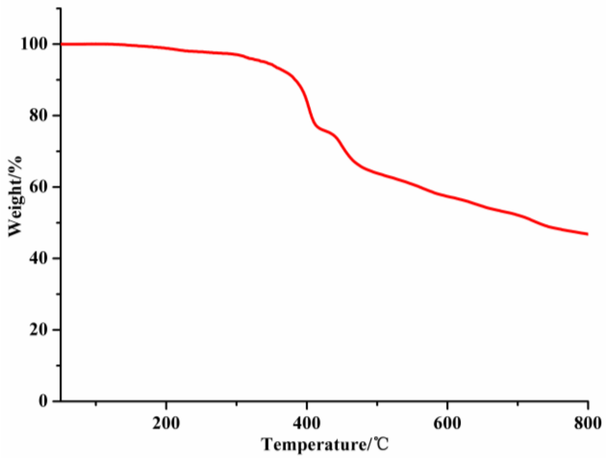

Figure S15. TGA of Pd@Y-DDQ.

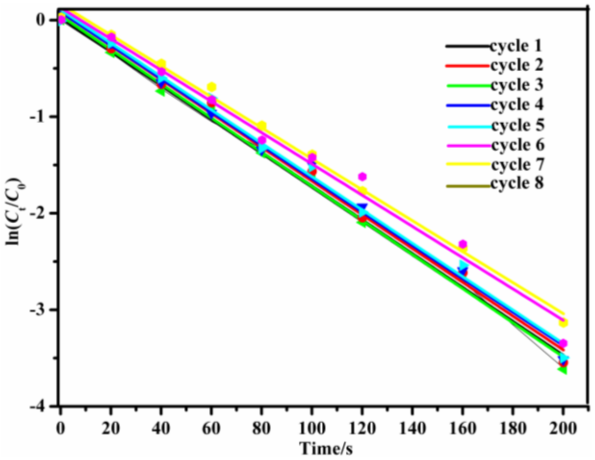

Figure S16. Relationship of ln(C_t/C_0) and reaction time t for 8 cycles of 4-nitrophenol reduction under the same reaction conditions over Pd@Y-DDQ catalysts.

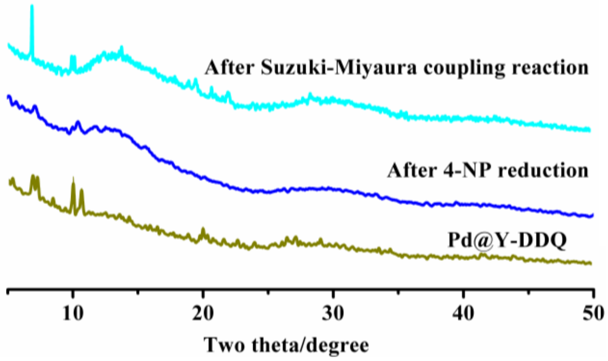

Figure S17. XRD patterns of Pd@Y-DDQ and the samples after catalytic reactions

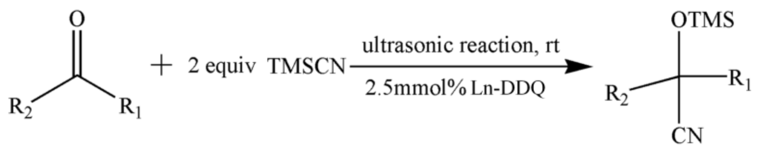

Figure S18. The cyanosilylation reaction in the presence of Ln-DDQ

Table S1. Crystal data and structure refinements for Ln-DDQs

Compound	Y-DDQ	Dy-DDQ	Eu-DDQ
Formula	$C_{103.68}H_{104.7}N_{22.23}O_{34.3}Y_3$	$C_{106}H_{114}N_{23}O_{33}Dy_3$	$C_{212}H_{228}N_{46}O_{66}Eu_6$
Formula weight	2477.71	2725.70	5388.16
Crystal size / mm	0.30×0.26×0.22	0.30×0.26×0.22	0.28×0.26×0.25
Crystal system	triclinic	triclinic	triclinic
Space group	$P\bar{1}$	$P\bar{1}$	$P\bar{1}$
a (Å)	12.465(3)	12.442(3)	12.594(3)
b (Å)	13.796(3)	13.727(3)	18.919(3)
c (Å)	17.733(3)	17.739(3)	25.394(3)
α (°)	107.73(3)	107.59(3)	105.53(3)
β (°)	93.52(3)	93.84(3)	92.86(3)
γ (°)	99.02(3)	98.66(3)	100.01(3)
D_c(g cm^{-3})	1.444	1.597	1.567
Z	1	1	1
F(000)	1274	1373	2728
Reflections collected	25565	26505	53959
Unique reflections	8815	9491	13894
μ (mm$^{-1)}$	1.604	2.047	1.716
Goodness-of-fit on F^2	0.918	0.921	1.040
R_1 [I>2σ(I)]	0.0656	0.0392	0.0921
wR_2 [I>2σ(I)]	0.1989	0.1290	0.3212
R_1 (all data)	0.0727	0.0436	0.1095
wR_2 (all data)	0.2069	0.1290	0.3546

Table S2. Selected bond lengths (Å) and angles (°) for Ln-DDQs

**Y-DDQ**					
Y1-O1	2.288(3)	Y1-O5	2.304(3)	Y1-O6	2.343(3)
Y1-O7	2.250(3)	Y1-O9	2.322(3)	Y1-O8	2.374(4)
Y1-O7	2.833(4)	Y1-O10	2.305(3)	Y2-O4	2.218(3)
Y2-O2	2.231(3)	Y2-O73	2.159(10)	Y2-O75	2.338(12)
O1-Y1-O5	80.15(12)	O7-Y1-O1	94.56(12)	O7-Y1-O5	157.54(13)
O7-Y1-O10	74.61(12)	O1-Y1-O10	76.57(12)	O5-Y1-O10	124.32(12)
O7-Y1-O9	83.91(12)	O1-Y1-O9	148.52(13)	O10-Y1-O9	132.18(12)
O4-Y2-O2	91.92(14)	O73-Y2-O2	92.5(3)	O4-Y2-O75	94.6(3)
O7-Y1-O8	78.32(14)	O9-Y1-O7	65.62(11)	O1-Y1-O8	75.20(14)
**Dy-DDQ**					
Dy1-O1	2.315(3)	Dy1-O3	2.364(3)	Dy1-O4	2. 814(4)
Dy1-O4	2.276(3)	Dy1-O5	2.318(3)	Dy1-O6	2.333(3)
Dy1-O7	2.305(3)	Dy1-O8	2.389(3)	Dy2-O40	2.209(4)
Dy2-O2	2.232(3)	Dy2-O9	2.237(3)	Dy2-O40	2.209(4)
O4-Dy1-O7	94.50(11)	O4-Dy1-O1	157.56(12)	O7-Dy1-O1	80.45(11)
O4-Dy1-O5	74.47(11)	O7-Dy1-O5	76.40(11)	O1-Dy1-O5	124.53(11)
O4-Dy1-O6	83.71(10)	O7-Dy1-O6	148.42(11)	O1-Dy1-O6	89.28(10)
O5-Dy1-O6	132.28(11)	O4- Dy2-O2	89.51(14)	O40-Dy2-O2	90.49(14)
O2-Dy2-O9	87.44(12)	O40Dy2 O2	89.51(14)	O5-Dy1-O8	138.06(11)
**Eu-DDQ**					

Eu1-O1	2.398(6)	Eu1-O3	2.342(6)	Eu1-O5	2.326(6)
Eu1-O6	2.308(6)	Eu1-O22	2.307(5)	Eu1-O24	2.406(6)
Eu1-O23	2.407(7)	Eu2-O2	2.334(5)	Eu2-O7	2.366(4)
Eu2-O11	2.327(4)	Eu2-O12	2.331(5)	Eu2-O26	2.299(5)
Eu2-O25	2.412(6)	Eu2-O27	2.410(5)	Eu5-O10	2.277(5)
Eu6-O9	2.257(6)	Eu5-O8	2.264(6)	Eu6-O4	2.288(5)
O22-Eu1-O6	157.7(3)	O22-Eu1-O5	74.3(2)	O6-Eu1-O5	124.3(2)
O22-Eu1-O3	95.2(2)	O26-Eu2-O11	95.13(16)	O6-Eu1-O3	78.8(2)
O5-Eu1-O3	78.0(2)	O26-Eu2-O12	157.36(19)	O11-Eu2-O12	79.02(18)
O26-Eu2-O2	73.83(18)	O12-Eu2-O2	124.85(18)	O11-Eu2-O2	76.76(18)
O93-Eu5-O8	77.3(13)	O93-Eu5-O8	102.7(13)	O93-Eu5-O8	102.7(13)
O9-Eu6-O4	94.7(2)	O9-Eu6-O34	112.2(4)	O4-Eu6-O34	105.4(4)
O9-Eu6-O34	112.2(4)	O26-Eu2-O7	84.14(16)	O11-Eu2-O7	148.18(18)

Table S3 Test of heterogeneity of the reaction

Catalyzed reaction		Stirring after filtration	
Time(min)	Yield (%)	Time(min)	Yield (%)
30	54	45	55

^{1}HNMR of the productions from Suzuki-Miyaura coupling reaction

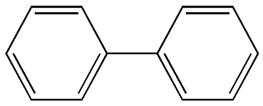

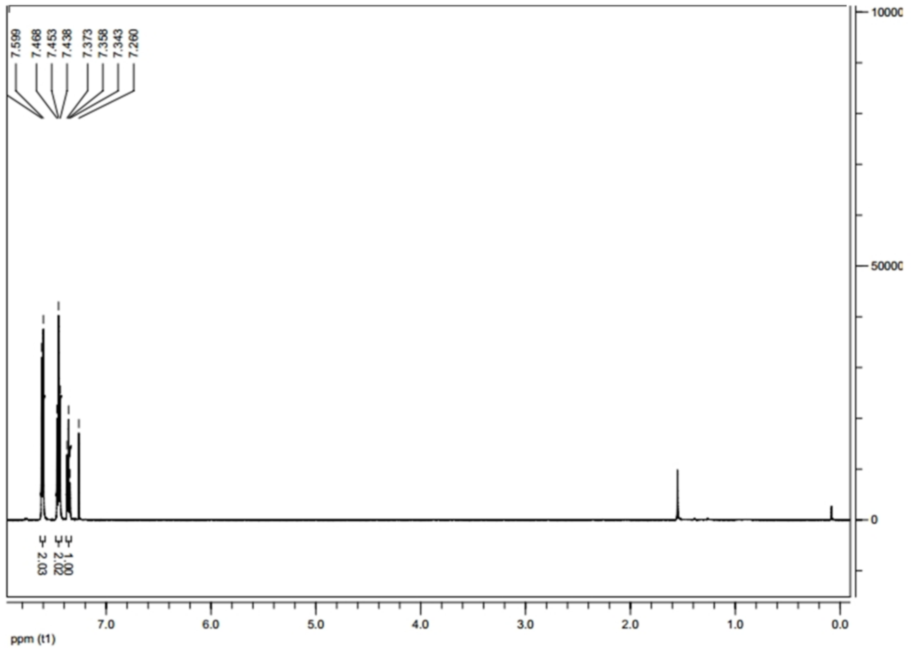

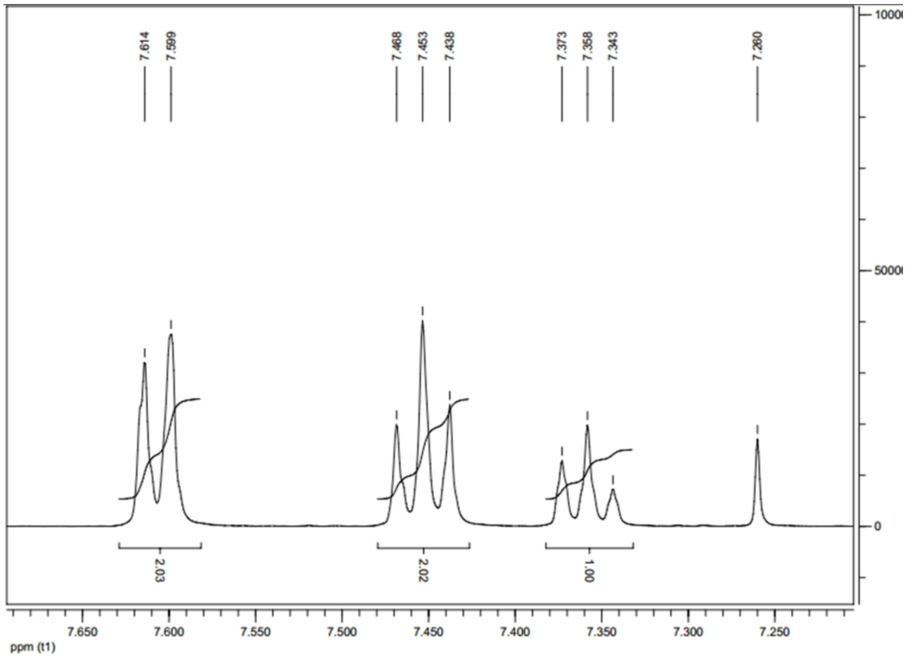

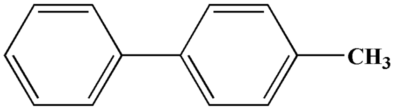

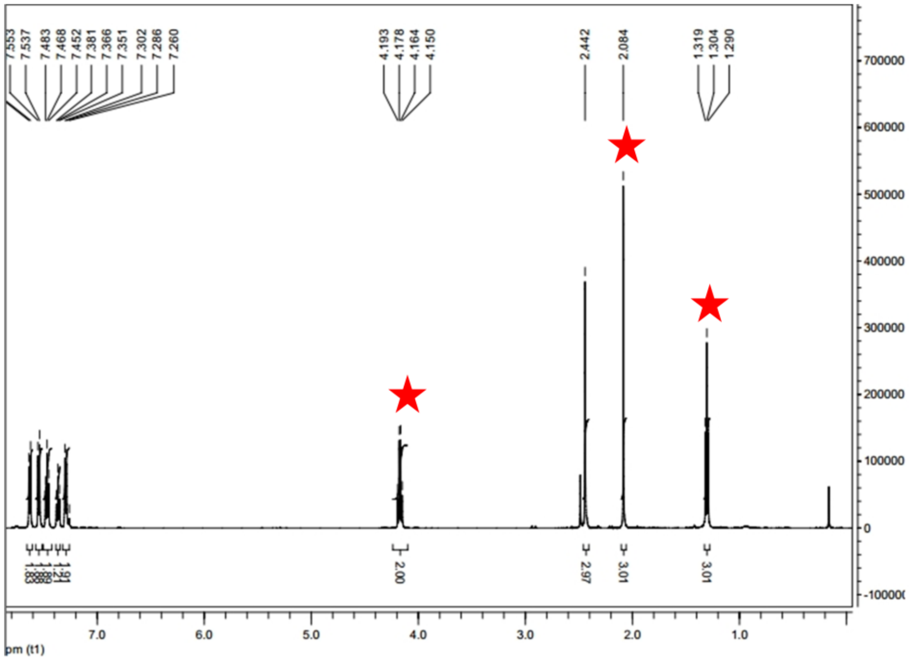

The red five-pointed stars in the spectrum indicate the existence of ethyl acetate.

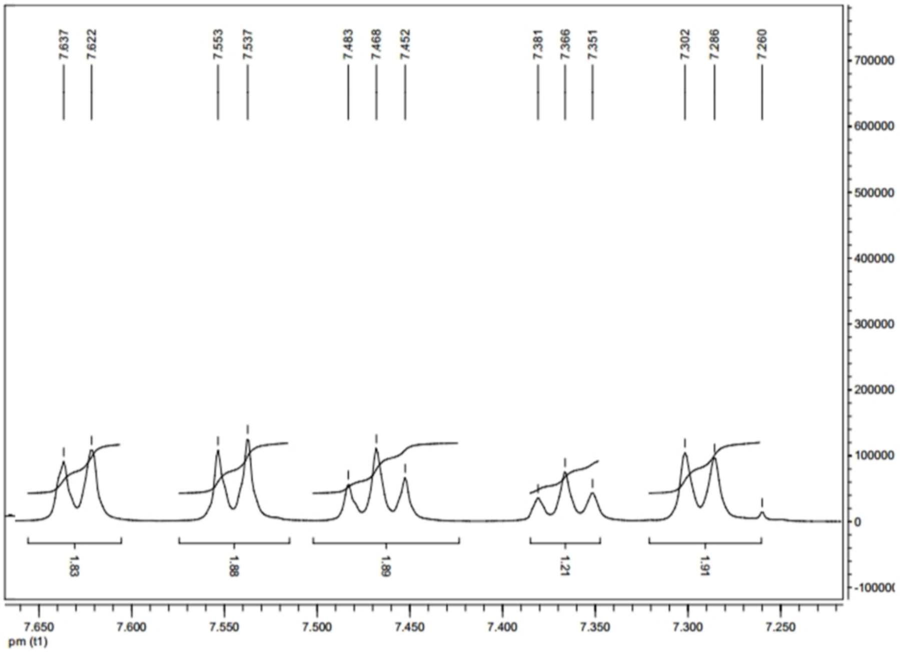

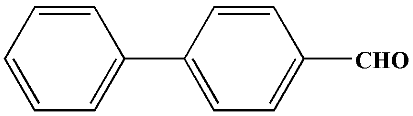
CHO

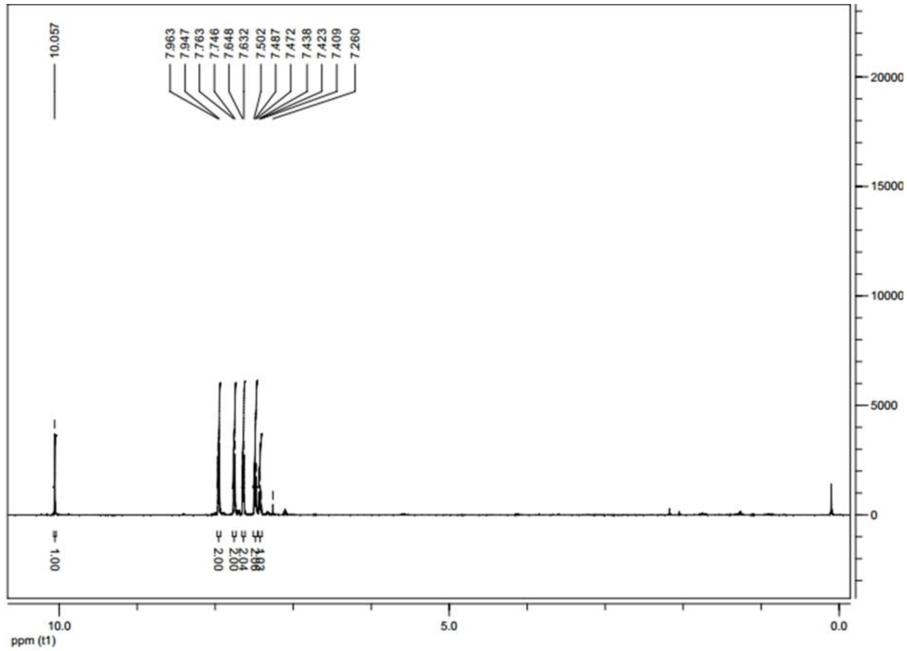
10.057
7.963
7.947
7.763
7.746
7.648
7.632
7.502
7.487
7.472
7.438
7.423
7.409
7.260
1.00
2.00
2.00
2.04
20000
15000
10000
5000
0
10.0
5.0
0.0
ppm (t1)

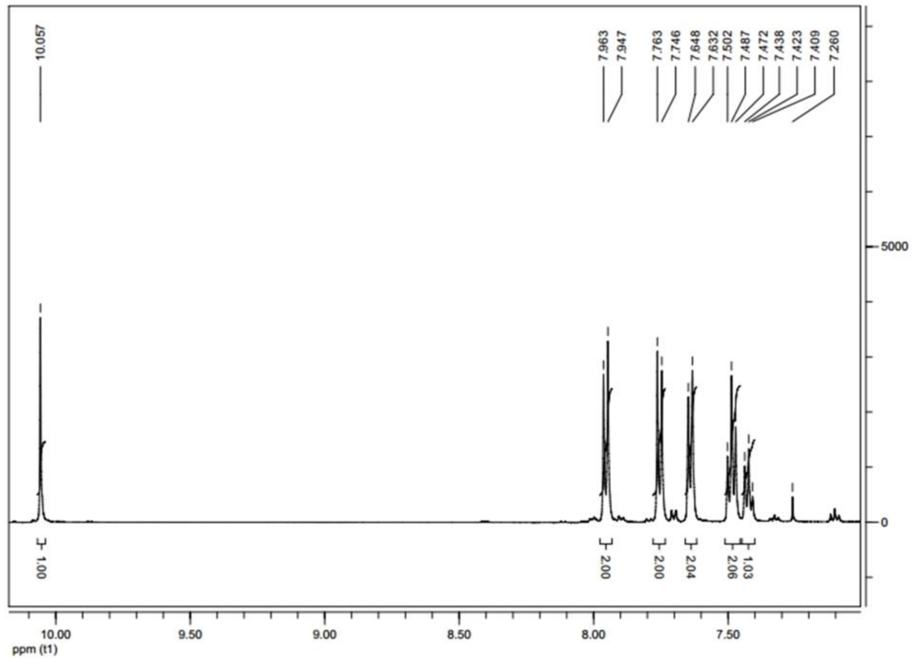
10.057
7.963
7.947
7.763
7.746
7.648
7.632
7.502
7.487
7.472
7.438
7.423
7.409
7.260
1.00
2.00
2.00
2.04
2.06
1.03
5000
0
10.00
9.50
9.00
8.50
8.00
7.50
ppm (t1)

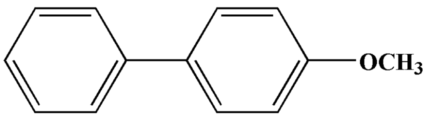

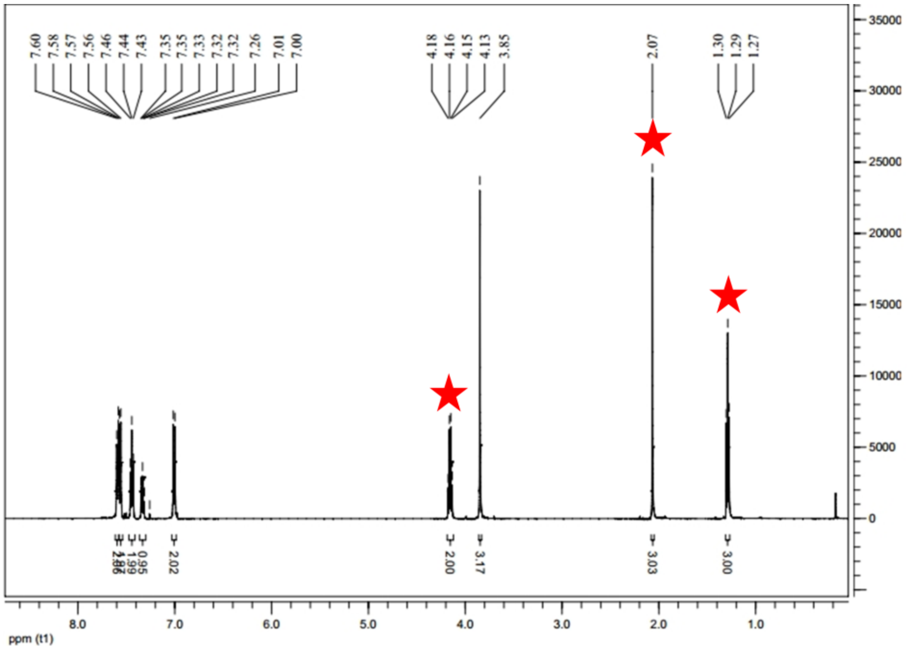

The red five-pointed stars in the spectrum indicate the existence of ethyl acetate.

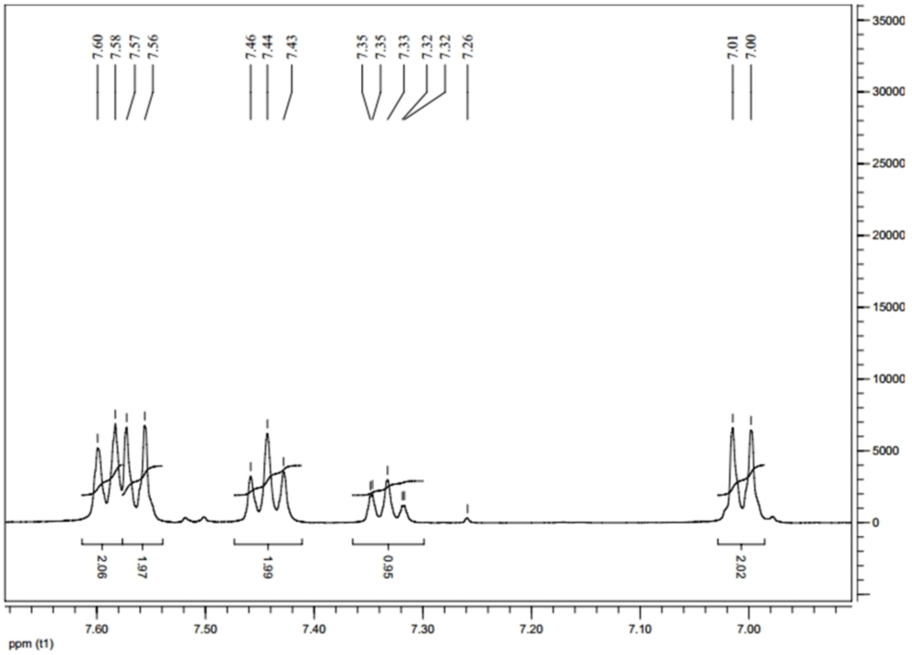

Supplement: Supplementary Information [file srep29728-s1.pdf]
